# The comparative study of skin staples and polypropylene sutures for securing the mesh in Lichtenstein’s tension-free inguinal hernia repair in HIV and hepatitis (B and C)-positive patients: a randomized controlled trial

**DOI:** 10.1097/MS9.0000000000001119

**Published:** 2023-07-31

**Authors:** Pragya, Anil Kumar, Manish, Rahul Ranjan, Nawal Kishore Jha

**Affiliations:** Departments of a General surgery; b Trauma Surgery and Critical Care, All India Institute of Medical Sciences, Patna; c Department of General Surgery, NMCH, Sasaram, India

**Keywords:** Hepatitis & HIV-positive, inguinal hernia, lichtenstein hernioplasty, NPIs, polypropylene sutures, skin staples

## Abstract

**Introduction:**

The mesh fixation in the Lichtenstein’s hernioplasty was traditionally performed with polypropylene sutures. A modification of this technique uses skin staples for securing the mesh. Using polypropylene sutures may increase the needle prick injuries (NPIs) in HIV and hepatitis-positive patients. This is the first study in India to compare the efficacy of anchoring the mesh with skin staplers and polypropylene sutures in hepatitis and HIV-positive patients.

**Methods:**

Fifty-two seropositive patients undergoing sixty repairs were randomized into two groups. In the control group polypropylene mesh was secured with polypropylene sutures and the skin was closed with ethilon. In the study group polypropylene mesh was secured with skin staples and the skin was closed with staples from the same stapler. Duration of surgery, postoperative complications, NPIs, recurrences and costs were compared.

**Results:**

The operation was significantly shorter when staples were used (median 46 vs. 57 min, *P*<0.00001). There was no significant difference in the incidence of postoperative complications. The NPIs was statistically significant in the suture group as compared to the stapler group (*P*<0.05).

**Conclusion:**

Using skin staples to secure the mesh in Lichtenstein inguinal hernioplasty significantly reduced the duration of surgery. It was as effective as conventional mesh fixation with polypropylene with added advantages to reduce the operating time, duration of exposure to infected blood, and the NPIs significantly in HIV, hepatitis B virus and hepatitis C virus-positive patients.

## Background

Inguinal hernia surgery is a commonly performed surgery at any healthcare centre^1^. The basic principle of inguinal hernia surgery is the tension-free closure of defect preferably by using mesh^2,3^. The conventional way to place the mesh over the defect is by polypropylene suture, which is a complex and important surgical step to prevent migration, wrinkling and curling the mesh^4^. In hernia surgery the needle prick injury (NPI) is very common (27%) hazard among surgeons which may transmit the HIV and hepatitis infection significantly^5^. The alternative way of polypropylene suture is skin staple to fix the mesh in the inguinal canal may be the effective method in HIV and hepatitis infected patients to prevent the NPIs in surgeons^6^. This trial was conducted first time in the country to assess the efficacy of using polypropylene suture and skin staples to fix the mesh in hernia surgery in HIV and hepatitis infected patients.

## Methods

This study was a single centeric, prospective randomized controlled trial conducted during 2018–2021. A total of 52 patients for 60 open inguinal hernia repair were included for the study. All the 60 inguinal hernia repairs were divided in two random groups. In each group, 30 repairs were done.This study was done as per the Institutional Ethical Committee norms. The institute ethics committee approved the study, vide letter number GNSU/NMCH/IEC/2018/RPEC/0654/2018/15.

All the HIV and hepatitis infected patients (Confirmed by laboratory test) with age ranges from 15 to 65 years and American Society of Anesthesiology (ASA) grade I and II, scheduled for open inguinal hernia surgery under spinal anaesthesia were included in the study. Patients with a history of recurrent inguinal hernia, complicated hernia, sliding hernia, patients with HIV and hepatitis negative report, paediatric inguinal hernia(age below 15 years), elderly patients (age more than 65 years), planned for laparoscopic surgery, patients allergic to drugs used in spinal anaesthesia, and ASA Grades III–V were excluded from the study. The sample size for the study was evaluated to be 60 considering duration of study and estimated incidence of HIV/hepatitis infected patients with inguinal hernia using the medical record data for such patients. All the enroled patients for open inguinal hernia surgery were sent to routine pre-anaesthetic check-up including all the basic investigations. Patient’s parts were prepared. An informed written consent was obtained from the participants about the study, it is randomization and treatment as per group allocation.

The study participants posted for open inguinal hernia surgery were randomly divided into two groups (30 in each group) using sealed envelope technique. In this technique, one envelope was picked up by the patient from a box which contains 60 envelopes in which thirty S Group(staples group) and thirty C Group (suture group) were mentioned. According to picked up envelope, the procedure Lichtenstein’s tension-free hernioplasty was planned to fix the mesh by skin stapes and polypropylene sutures in the study group (S) and control group (C), respectively. In this study both the participants and researchers were aware about the nature of treatment as per open label randomized trial. All the members of surgical and anaesthesia team were aware to follow the universal precaution strictly. One day prior to surgery, the re-evaluation of all investigations report, pre-anaesthetic check-up and part preparation were done. The patient was kept on fasting overnight. On the day of operation, one preoperative dose of tetanus toxoid and antibiotics was given. After induction of spinal anaesthesia, the patient was handed over to surgical team to proceed the surgery. The hernia sac was identified and reduced after opening the inguinal canal. Once the cord was isolated and the hernial sac reduced, the mesh was placed and fixed with polypropylene sutures and skin staples in the control (C) and study group (S), respectively. In study (S) group, the mesh was fixed with multiproximate skin staple , one staple at pubic tubercle, four staples along with inguinal ligament, four staples over conjoint tendon and two staples lateral to the deep inguinal ring. The external oblique aponeurosis was closed with vicryle 2-0. In the control group (C), mesh was fixed with the polypropylene suture 1-0 suture first at pubic tubercle followed by at inguinal ligament (three to four stiches), conjoint tendon (three to four stiches) and finally one stich lateral to the deep inguinal ring. The external oblique aponeurosis was closed with the polypropylene 2-0. In the study group the skin was a closed with skin staples while in the control group interrupted sutures of Ethilon 2-0 used. The surgery for all 60 open inguinal hernia repair was performed by a professor and his team with 14 years of experience in General surgery Department. All repairs in both the group were performed by same team in view to avoid bias on the impact of the result.

The primary outcomes were duration of surgery, NPI and transmission of hepatitis and HIV infections to surgeons. The cost of surgery and postoperative complications were considered as secondary outcome.

**Figure 1:**
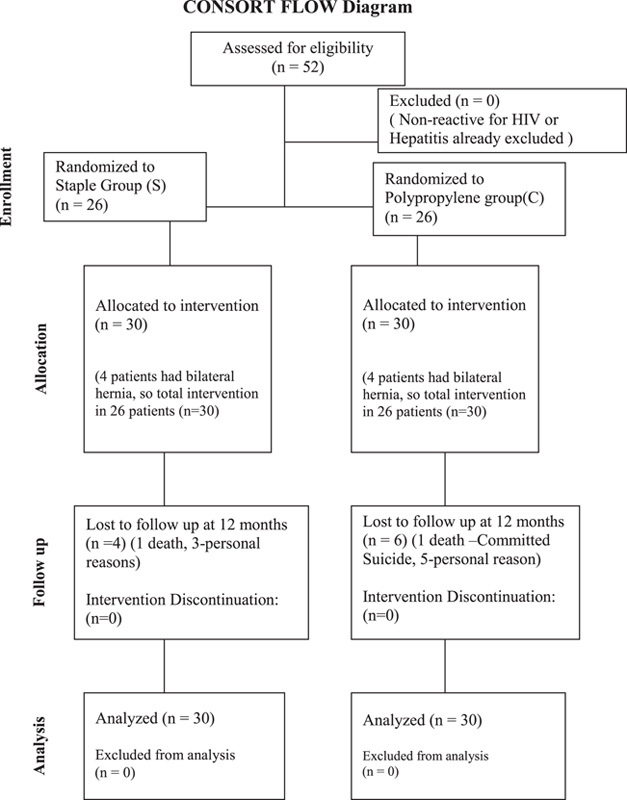
CONSORT FLOW Diagram.

The demographic data, duration of surgery (Time period for skin incision to the beginning of mesh fixation and mesh fixation to completion of the skin closure), postoperative complication, NPI and the cost of surgery for both the group were recorded. The patients were discharged either after 6–8 h of surgery or on the next day. All patients were called for follow-up in outpatient clinic after 7 days of surgery for the wound evaluation and stiches removal. The patients were further follow-up at 1 month, 3 months, 6 months and 12 months and then at every 6 months interval for the clinical evaluation for the morbidity and mortality related to surgery as well as HIV and hepatitis infections. A CONSORT flow diagram related to enrolment, allocation, intervention, follow-up and analysis of patients is depicted in Figure 1.

### Statistical analysis

All the data were collected in pre-formed data capture sheets and evaluated. Data were described in terms of range; mean±SD, median, frequencies (number of cases) and relative frequencies (percentages) as appropriate. A descriptive analysis for all possible variables was performed. For comparing categorical data, χ^2^ test was performed, and the fisher exact test was used when the expected frequency was less than 5. A *P* value of less than 0.05 was considered statistically significant. For non-parametric data, the Mann-Whitney U test was applied. For continuous data, an independent *t*-test was applied. A one-way ANOVA test was applied for subgroup analysis. All statistical calculations were done using SPSS (Statistical Package for the Social Science), a version 22 statistical program.

## Results

### Demographic and clinical profile

Out of 52 HIV/hepatitis infected patients enroled in the study, the majority were from the fourth to fifth decade of life (Table 1). The average age of the patients, who participated in this study was 44.3±3.3 years. The majority of the patients were male (94.23%) (Table 2). Out of 52 enroled patients, the diagnosis of hernia on right side in 28 patients, on left side in 16 patients, and bilateral side in 8 patients was made (Table 3). The most common presenting symptom was swelling (66.4%), followed by pain in the in groin region (26.2%), the rest of the patients presented with discomfort in the scrotoinguinal area (7.4%).

**Table 1:** Age wise distribution of the study participants.

Age group (year)	Staple group (S)	Polypropylene group ( C)
16–25	00	04
26–35	01	01
36–45	09	10
46–55	09	04
56–65	07	07
Total	26	26

**Table 2:** Sex-wise distribution.

	Staple group (S)	Polypropylene group (C)	Total
Male	26	23	49
Female	00	03	03
	26	26	52

**Table 3:** Right, left or bilateral inguinal hernia in staple and polypropylene group.

Side of inguinal hernia	Staple group (S)	Polypropylene group (C)
Right side	12	16
Left side	10	6
Bilateral	4	4

The mean duration of symptoms was 15±11.03 months, the minimum duration was 1 month, and the maximum duration was 40 months. In our study, 12% of patients were on antiviral (HIV/hepatitis) treatment, 11% of patients were hypertensive, 10% patient with chronic kidney diseases, and 9.7% had type 2 diabetes mellitus. Out of 52 enroled patients, 40 patients were reactive to hepatitis and remaining 12 for HIV. A family history of HIV/hepatitis diseases was present in 20% of the patients. The 52 HIV/hepatitis infected patients with 60 inguinal hernia repairs were randomized to study (S) and control (C) group for the fixation of mesh Lichtenstein’s hernioplasty. The maximum numbers of patients (*n*=19) were in the age group of 36–45 years. In our study 50 patients were male and 2 patients were female. Both the females were allocated in the polypropylene suture group. In our study out of 60 inguinal hernia repairs, 11 were direct inguinal hernia while 49 were indirect inguinal hernia.

### Duration of surgery

The duration of surgery for all 52 patients operated for Lichtenstein’s hernioplasty was calculated (Table 4). The median duration of surgery from the beginning of the mesh insertion to the end of the operation in staple group (S) was 46±4.25 min as compared to 57 ± 6.15 min in polyprolene group (C). The difference in duration of surgery from the start of surgery to insertion of mesh was not significant (*P* value=0.45) between both the groups, but the difference in duration of surgery from mesh insertion to the completion of surgery was significant (*P* value<0.00001). The difference in total operative time between the two groups was also significant (*P* value<0.00001).

**Table 4:** Comparison of duration of surgery in both the groups.

	A (Mean±SD)	B (Mean±SD)	C (Mean±SD)
Staple group (*n*-30)	32.39±3.65	13.71±2.24	46.10±4.25
Polyprolene group (n-30)	33.24±4.97	24.14±2.82	57.40±6.15
	t=−0.76, *P*=0.45 not significant	t=15.92, *P*<0.00001 significant	t=8.33, *P*<0.00001 significant

A: Duration from start of surgery to mesh insertion, B: Duration of mesh insertion to completion of surgery, C:Total operative time.

### Postoperative complications

The postoperative complications including swelling, induration, seroma formation, mesh infection, wound haematoma, recurrences at 12 months and NPI is summarized in (Table 5). There was no statistical differences for postoperative complications in both the groups except for NPI which was statistically significant (*P* value<0.001) in polypropylene suture group.

**Table 5:** Comparison of complications in both the groups.

Complications	Staple group	Polypropylene group	Total	*P*
Needle prick injury	0	6	6	<0.05
Induration and Swelling	8	7	15	0.278
Seroma	4	5	9	0.096

### Cost comparison in stapler and polypropylene group

In our study, an average operative cost of Lichtenstein’s mesh hernioplasty for unilateral inguinal hernia in stapler group was 34.49 dollar while in polypropylene suture group it was 31.71 dollar. There was no significant difference between the cost of surgery in two groups (*P* value=0.18). The operative cost for Lichtensteins mesh hernioplasty for bilateral inguinal hernia in stapler group was 52.56 dollars, while in polypropylene suture group, it was 51.84 dollars. There was no difference between the cost of surgery in two groups (*P*>0.05) (Table 6).

**Table 6:** Comparison of cost of the surgery in both the groups.

Cost	Staple group	Polypropylene group
Unilateral inguinal hernia repair	34.49 Dollars	31.71 Dollars
Bilateral inguinal hernia repair	52.56 Dollars	51.84 Dollars

### Follow-up

Most of patients (22 patients of study and 24 patients of control group) were discharged after 6–8 hours of surgery, 4 patients of study and 2 patients of control group on the next day of operation. All patients were come to outpatient clinic of general surgery unit after 7 ± 3 days for stiches removal. Out of all 52 operated patients, 3 patients of study group and 4 patients of control group developed stich site infection which was managed conservatively. At 3 months and 6 months of follow-up, all patients were doing well. At 12 month’s follow-up, 10 patient (6 from control and 4 from study group) were lost to follow-up because of personal and economical problems which was conveyed telephonically. Unfortunately two patients (one from each group) died at home (one patient committed suicide from control group). The reason of death in second patient was unknown as told by his relative.

## Discussion

The present study was conducted in department of Surgery from 2018 to 2021. Our results with the technique of using skin stapler for mesh fixation in comparison with the conventional method of mesh fixation using polypropylene suture in HIV and hepatitis B positive patients has been proven superior in term of reducing the transmission of blood born diseases. The age of the patient in the stapler group and polypropylene group ranged from 16 to 65 year. The two groups were statistically comparable in relation to age (*P*>0.05). There were 49 male and 3 female (all in polypropylene group) patients in the presented study. One of the most commonly performed surgeries in any surgical unit is hernia surgery. Initially the concept of tissue repair was in practice for hernia surgery and later on the concept of tension-free repairs with mesh considered as gold standard treatment for the hernia repair. The use of skin staples for fixing the mesh in inguinal hernia surgery was first time performed by Egger *et al.* ^7^. Since then many studies has been conducted and found that the use of skin staples in the Lichtenstein inguinal hernia repair significantly reduced the duration of the surgery as compare to conventional polypropylene suture^8–11^. As HIV is the third widespread epidemic in the world causing huge burden in the developing countries like India and estimated to 2.1 million people living with the disease^12^. The prevalence rates of hepatitis B virus in India is 2% to 4% and has 40 million hepatitis B virus carriers and are prone to develop cirrhosis and premature death^13^.

The incidence of hepatitis B and HIV in the state where study was done is 5% and 0.91%, respectively, as per local newspaper. The surgeons and their assistant are always on the risk to get NPI more commonly in abdominal wound closure (52%) followed by hernia surgery (27%)^5^. Considering all these facts, we compared a study (staple group) in which skin staples were used for anchoring the mesh with the control group (polypropylene suture group). In our study the result showed that the NPI was more common in hernia surgery while using conventional polypropylene suture as compared to skin staples. Mills *et al.* ^8^ conducted the similar study and found that the operating time was significantly shorter in staples group as compared to polypropylene suture group (median 20 vs. 29 min with significant *P* value<0.01) . In another study which was conducted by P Garg Chaitanya *et al.* ^14^ reported that the operating time was significantly shorter with the use of staple in securing the mesh ( median 42 min 30 s vs. 54 min 30 s, *P* value<0.01). There was no significant difference in the incidence of postoperative complications or pain or recurrences in either group in the follow-up period similar to present study. In our study the median length of operating time was 11 min shorter in staple group (*P*<0.00001) as compared to polypropylene group while this difference was 9 min in case of Mills *et al.* ^8^ study. As per Mills, the shorter operating time may be associated with a reduced risk of wound infection, keep the lowest risk of anaesthesia. The surgeons and their assistant are at risk of accidental NPIs. NPIs are considered as severe occupational health hazards worldwide and its prevalence among healthcare workers was between 57 and 73%^15,16^. Operation rooms were one of the common areas where NPIs occurred frequently to surgeons and their assistant^17,18^. One more advantage of using staple for fixation of mesh is that there is no chance of NPIs, which is comparatively more associated with the polypropylene suture fixation. The use of skin staple was especially important in our study where all study participants were HIV and hepatitis-positive. The use of skin stapler for fixation of mesh reduces the operating time and consequently reduces the duration of exposure to infected blood as well as it reduces the NPIs. Our study showed that it is safer to fix mesh with staples in HIV and hepatitis-positive cases. The main complications in our study were swelling and wound infection (25%), NPI (20%) exclusively in polypropylene suture group and seroma formation (15%).

There was significant difference for NPIs complication in staple group as compared to suture group. None of the patients developed haematoma, mesh infection and wound gaping like other studies^8,14^. Chaitanya and colleagues also had reported similar results with wound gaping in one patient^14^. Mills^8^ had reported haematoma in four patients unlike our study. The major concerns with the use of skin staples reported in the previous studies^19–22^ were increased risk of wound infection, entrapment neuropathy, and potential vascular injury, but none of these complications occurred in our study.

### Limitations of the study

The prospective multicenteric trial considering large sample size with long term follow-up for patients for each group is required to validate these findings in order to recommend the definite guidelines.

## Conclusions

Although the multicentric trial with large sample size study is required to validate the findings but the stapler is more acceptable method to fix the mesh in hernia surgery as compared to polypropylene suture in terms of significant reduction in operative time. This is also considered as a safer technique in HIV and hepatitis-positive patient as it avoid NPIs and reduces the transmission of HIV and hepatitis infections among the HCWs.

## Ethical approval

This study is as per Institutional Ethical Committee norm. The institute ethics committee approved this study, vide letter number GNSU/NMCH/IEC/2018/RPEC/0654/2018/15.

## Consent

Written informed consent was obtained from the patient for publication of this case report and accompanying images. A copy of the written consent is available for review by the Editor-in-Chief of this journal on request.

## Sources of funding

None.

## Author contribution

Conception or design of the work: A.K. and Pragya. Data collection: Manish and N.K.. Data analysis and interpretation: R.R. and Pragya. Drafting the article: A.K. and Pragya. Critical revision of the article: Manish, N.K.J. and Pragya. Final approval of the version to be published: all authors. The author(s) read and approved the final manuscript.

## Conflicts of interest disclosure

The authors declare that they have no conflicts of interest.

## Research registration unique identifying number (UIN)

None.

## Guarantor

Dr Anil Kumar.

## Data availability statement

The data that support the findings of this study are available from the corresponding author, Dr Anil Kumar, upon reasonable request.

## Provenance and peer review

Not commissioned, externally peer-reviewed.
